# Concordance for changes in allergic asthma domain variables after short-term corticosteroid therapy

**DOI:** 10.1186/s12890-020-1166-2

**Published:** 2020-05-14

**Authors:** Philip E. Silkoff, Mark Sarno, Solomon Ssenyange, Vivek Balasubramanyam, Brian Awabdy, Ryan Leard

**Affiliations:** 1 Philadelphia, USA; 2 Vision Clinical Research, 1501 San Elijo Road, Suite 104, #213, San Marcos, CA 92078 USA; 3 Spirosure Inc, 7020 Koll Center Pkwy #110, Pleasanton, CA 94566 USA

**Keywords:** Asthma, Exhaled, Nitric, Oxide, Domains, Concordance, Corticosteroids

## Abstract

**Background:**

Asthma is a complex syndrome with multiple domains including symptoms, lung function, asthma control, and airway inflammation. A study of Fenom PRO™, a novel monitor for exhaled nitric oxide (FeNO), provided an opportunity to look at concordance/discordance (C/D) for changes in multiple asthma domains over a 2-week period after corticosteroid therapy.

**Methods:**

Non-steroid-treated adults and children with uncontrolled asthma had asthma domain measures, (FeNO), forced expired volume in 1 s (FEV_1_), the 6-item Asthma Control Questionnaire scores (ACQ6), and daily asthma symptoms, assessed before and after a 2-week course of corticosteroids. Asthma symptoms were assessed using a custom novel twice-daily symptom scale (ASX). C/D bidirectional changes in all domains were calculated around both the zero point, and around the minimal important difference (MID) in relevant subjects.

**Results:**

There was a highly significant fall in mean FeNO of 51.7% over 2 weeks (*p* < 0.0001) accompanied by significant improvements in mean FEV_1_, ACQ6 and ASX scores. However, C/D between individual domains varied considerably between subjects. The C/D between parameters for any change around zero for the combined adults and pediatric population was best for FeNO and ACQ6, 79.3/20.7% while FEV_1_ was more discordant than other parameters in general. When considering changes around the minimal important difference (MID) in a subset, the level of concordance increased in general, with FeNO and ACQ6 demonstrating a C/D of 93.5/6.6%.

**Conclusion:**

This data demonstrates that the concordance between changes in the asthma domains is often substantially less than 100%. Reasons for this may include different time courses for change of the separate domains, the degree of abnormality for each domain at baseline, as well as intrinsic limitations of each parameter.

## Background

Asthma is a complex syndrome characterized by episodic and chronic symptoms e.g. wheezing, cough, chest tightness and dyspnea, as well as periodic exacerbations which may require short courses of corticosteroids. The asthma syndrome can be regarded as a constellation of domains including symptoms with inherent subjectivity, lung function e.g. the forced expired volume in one second (FEV_1_), airway inflammation commonly assessed by exhaled nitric oxide (FeNO), and asthma control as assessed by the asthma control questionnaire (ACQ), all of which may be disturbed to different degrees in an individual subject.

Of interest, dynamic changes in asthma domains, either spontaneous over time or as a result of therapeutic intervention e.g. with corticosteroids in these domains may not be concordant. For example, poor perceivers can experience declines in lung function without any increase in accompanying symptoms.

A recent study was performed to fufill US regulatory requirements [1] for clearing a novel FeNO monitor Fenom Pro™. The clinical precision, accuracy and comparison to NIOX VERO®, a commonly used FeNO monitor was recently reported [2]. Adults and children with uncontrolled asthma were treated with corticosteroids over a 2-week period. This study provided an opportunity to evaluate short-term concordance/discordance (C/D) between asthma domains, namely day and nighttime symptoms, FEV_1_, asthma control via the 6-item ACQ6, and airway inflammation using FeNO which is the focus of this report.

## Methods

The study was conducted at multiple physician offices located in the USA. Based on US regulatory requirements to register a novel device, the primary objective was to demonstrate that a novel FeNO monitor, Fenom PRO™ (Spirosure, Pleasanton, CA, USA), can detect a significant change in FeNO, a marker of airway inflammation following short-term corticosteroid therapy prescribed for uncontrolled asthma. A secondary objective was to demonstrate that the change in FeNO after corticosteroid therapy was accompanied by improvement in FEV_1_, asthma symptoms, and the 6-item asthma control (ACQ6) scores. The study was approved by the BioMed institutional review board on December 8, 2016. The focus of this report is an evaluation of concordance for separate asthma domains in the study.

### Patient and public involvement

There was no patient or public involvement in developing this protocol as all subjects were screened and recruited on the same day. No patients were involved in helping recruit this study and the results have not been communicated to the study participants.

### Study design and duration

**Fig. 1:**
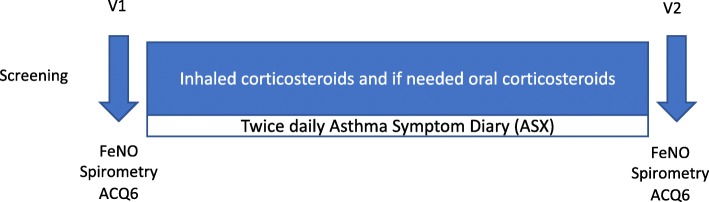
The Study design consisting of two visits, V1 and V2. FeNO, spirometry and ACQ7 scores were assessed before and after 2 weeks of corticosteroid therapy. Daily symptoms were collected with the twice-daily asthma symptom diary (ASX) between V1 and V2

This was an open label, non-randomized, prospective, study evaluating FeNO, spirometry, asthma control and asthma symptoms in non-steroid-treated adult and pediatric subjects with uncontrolled asthma before and after 2-week corticosteroid treatment (see Fig. 1). The study consisted of two visits, V1 and V2, separated by a period of approximately 14 days. Subjects were started on high dose inhaled corticosteroids (ICS) with the addition of oral corticosteroids if clinically indicated. A safety follow-up telephone call occurred on Day 21 ± 3 such that the total duration of the study was a maximum of 24 days.

### Study population

The study population comprised male and female subjects aged 5 years and older spontaneously presenting with uncontrolled asthma based on recent symptoms that required intervention. No specific etiology for the uncontrolled asthma is available. No asthma controllers were permitted including corticosteroids or leukotriene-modifying agents for 2 weeks and biologics for 12 weeks prior to enrolment. Subjects who were not already treated with a short-acting bronchodilator (SABA) were provided with such at Visit 1. The subjects were required to have a FeNO level > 25 parts per billion (ppb) (children < 18 years of age), or > 30 ppb (adults ≥18 years old) at Visit 1, increasing the probability for an allergic asthma phenotype. This eligibility criterion for a raised FeNO is driven by regulatory requirements to show that a novel FeNO monitor can detect a reduction in FeNO after corticosteroid therapy. Subjects were excluded if they were cigarette smokers within the past 6 months, presented with a ≥ 10 pack-year history of smoking, had other respiratory conditions, or had other clinically significant medical conditions.

### Primary endpoint

The primary endpoint was the % change from baseline from Visit 1 to Visit 2 in FeNO.

### Secondary endpoints

Secondary endpoints included the change from baseline from Visit 1 to Visit 2 pre-bronchodilator FEV_1_, ACQ6 or the pediatric ACQ6 (pACQ6), and total day and nighttime asthma symptoms (TASX) measured with a custom-built questionnaire for this study, the ASX, to be completed twice-daily by adult subjects and caregivers for pediatric subjects.

### Study procedures

**Fig. 2:**
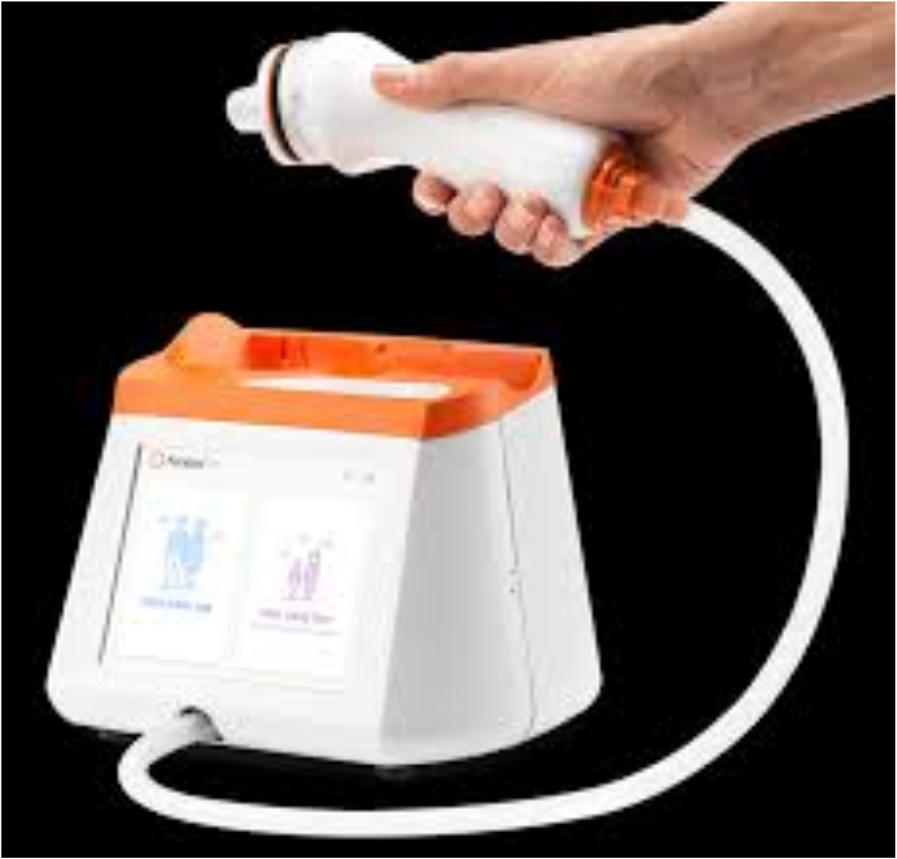
The Fenom PRO device showing the handheld mouthpiece and mouth filter

FeNO was measured using the recently-cleared Fenom PRO™ (see Fig. 2; Spirosure, Inc., Pleasanton, CA, USA) [2]. Fenom PRO simplifies the measurement process and provides rapid results in less than 30 s (s). Fenom PRO™ monitors and encourages users to achieve an expiratory flow rate of 50 ml/s as recommended by the American Thoracic Society (ATS) guidelines from 2005. Subjects inhaled ambient air to total lung capacity, placed their lips on the mouthpiece, and exhaled while targeting a flow rate of 50 ml/s assisted by visual and auditory feedback indicating the actual exhalation flow rate versus the desired flow rate. The Fenom PRO™ device indicates whether the required exhalation flow rate parameters were met. Repeated exhalations were allowed to achieve 2 valid exhalations.

Spirometry was measured using each clinic’s spirometers that conformed to ATS guidelines.

Asthma control was captured using the ACQ6 and pediatric ACQ6 [3–5] before other study procedures.

Twice daily symptoms were captured using a custom-built novel symptom diary, the ASX that surveyed asthma symptoms, namely wheezing, chest tightness cough and dyspnea as well as nighttime awakenings due to asthma. The ASX consists of five symptom questions regarding wheezing, coughing, tightness of the chest, breathlessness, and activity limitation during the day and four symptom questions wheezing, coughing, tightness of the chest, breathlessness during the night each scored from 0 to 4 making a total potential score (TASX) of 36 for day and night together. There is no validated minimal important change (MID) for the TASX.

### Statistical methods

The primary endpoint, the % change from baseline (V1 to V2) in FeNO was analyzed via a two-sided paired t-test or Wilcoxon Signed Rank test, depending on the distribution of the data, at the 0.05 level of significance. The absolute changes for FEV_1_, ACQ6 and TASX were analyzed using the same method. The TASX scores were averaged over Day 1 and 2 after Visit 1 and the last 2 days before Visit 2 for the purpose of assessing change from Visit 1 to Visit 2. Investigation was made of the association of changes in all asthma domains by calculating correlation coefficients.

The concordance/discordance (C/D) was calculated for each pair of study outcomes (FeNO, ACQ6, FEV_1_, and TASX) using bidirectional changes (improving or worsening of asthma as indicated by each outcome) of any magnitude around their respective zero points and also the published minimal important differences (MID) for FEV_1_ (100 mls.) [6], ACQ6 (0.5) [5], and a significant change for FeNO of at least 10 ppb based on the ATS recommendations from 2011 [7].

## Results

The study was performed at 10 investigational sites in the USA. The study was approved by the BioMed IRB and all subjects or caregivers for minors underwent an informed consent procedure and signed consent forms and assent forms for minors.

### Subjects disposition

Of 128 screened subjects, 85 enrolled into the study and 84 comprised the intent to treat population (ITT). Table 1 presents demographic variables for the ITT population (*n* = 84). All subjects were started on high dose ICS therapy while only three subjects were also started on OCS. Based on self-reported compliance in the daily diaries, 84 of 85 subjects complied with corticosteroid therapy started at V1. Only 1 subject reported not taking corticosteroid therapy for the entire duration of the study.

**Table 1:** Demographics and relevant medical history for the ITT population at baseline, V1

**Age (years)**	
n	84
Mean	28.9
SD	19.48
Median	19.5
Min, Max	8, 79
**Age, n (%)**	
5–11 years	12 (14.3)
12–17 years	26 (31.0)
> =18 years	46 (54.8)
**Sex, n (%)**	
Male	48 (57.1)
Female	36 (42.9)
**Race, n (%)**	
Caucasian	60 (71.4)
Black or African American	13 (15.5)
Asian	4 (4.8)
American Indian or Alaskan Native	0
Native Hawaiian/Pacific Islander	0
Two or more races	3 (3.6)
Other	4 (4.8)
**Ethnicity, n (%)**	
Hispanic or Latino	19 (22.6)
Not Hispanic or Latino	64 (76.2)
Unknown/Not Provided	1 (1.2)
^a^Food allergy	30.1%
^a^Allergic rhinitis	18.1%
^a^Atopic dermatitis	3.5%
Asthma age of onset mean (SD)	8.4 (11.8)
Asthma duration mean (SD)	18.4 (15.9)

^a^based on commonest term captured in the medical history at screening

### Asthma characteristics at baseline

Table 2 presents baseline asthma characteristics for the ITT population. In general, this was a mild population based on a mean % predicted FEV_1_ of 82.7% although subjects had a wide FEV_1_ range (26.8–139.5%), and on average had markedly uncontrolled asthma, with a mean (95%CL) ACQ6/pACQ6 of 2.06 (1.83–2.29) where uncontrolled asthma is defined as a score > 1.5.

**Table 2:** Asthma characteristics at baseline, for the ITT population at V1

**FEV**_**1**_**(L)**	
n	84
Mean	2.676
SD	0.9115
Median	2.675
Min, Max	0.79, 5.11
**FVC (L)**	
n	84
Mean	3.625
SD	1.2339
Median	3.710
Min, Max	1.40, 6.82
**FEV**_**1**_**/FVC**	
n	84
Mean	74.355
SD	10.4398
Median	74.164
Min, Max	52.72, 120.00
**% Predicted FEV**_**1**_	
n	84
Mean	82.72
SD	17.250
Median	83.75
Min, Max	26.8, 139.5
**ACQ6 and pACQ6**	
n	84
Mean	2.056
SD	1.033
Median	1.833
Min, Max	0.17, 5.33

### Primary endpoint

**Fig. 3:**
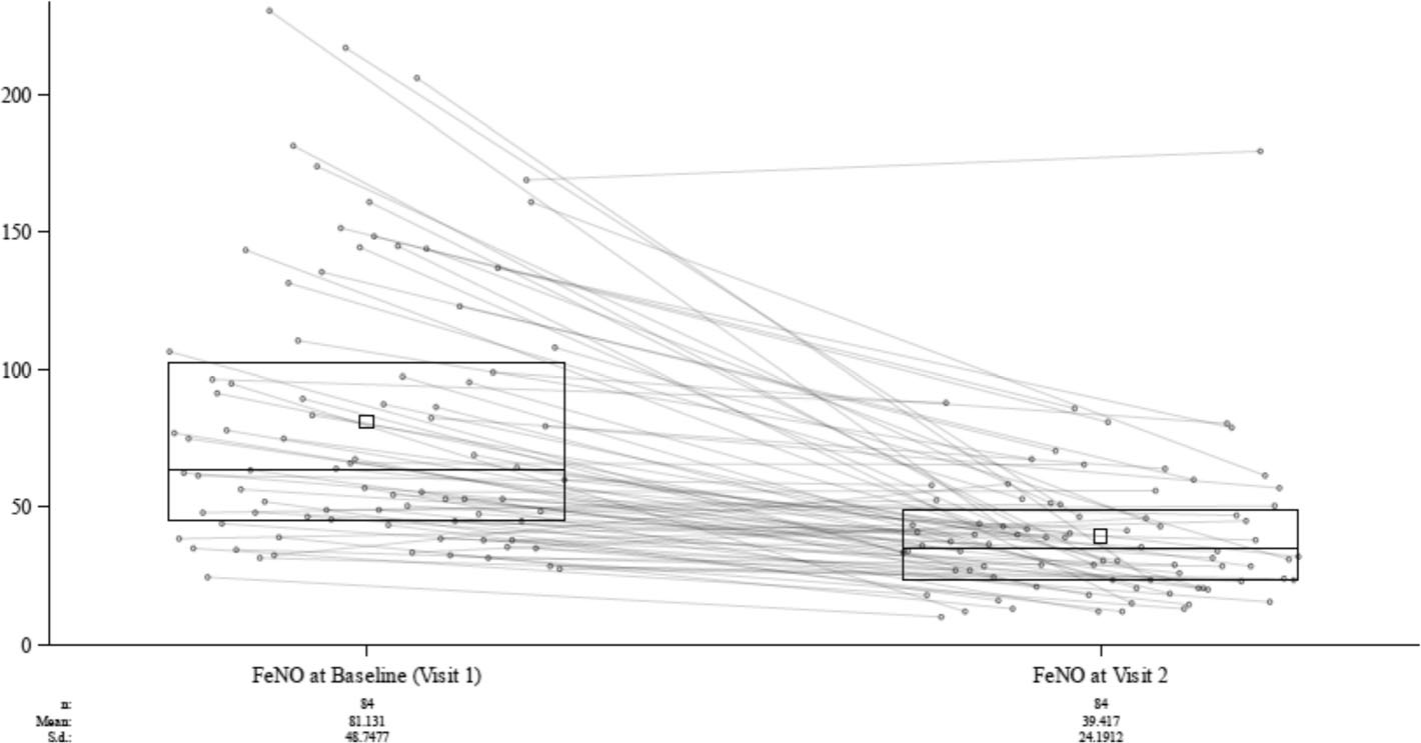
A box plot of FeNO values (ppb) at Visit 1 and Visit 2 for the ITT population with overlaid line plotting of all subjects in the ITT population. The lower and upper bounds of the boxes represent the 25th and 75th percentiles of the distributions (the interquartile range) and the internal box symbols and horizontal lines within each box represent the mean and median values, respectively. The figure indicates the diminution of FeNO values across the population as a result of corticosteroid therapy

For the primary endpoint in the ITT population, the % change in FeNO from Visit 1 to 2, was − 51.4% (absolute change − 41.7 ppb), *p* < 0.0001 as shown in Table 3. Figure 3 presents a box plot of FeNO values (in absolute ppb) at Visit 1 and Visit 2 with overlaid line plotting for each individual subject indicating the change of FeNO values from V1 to V2. As shown, FeNO values decreased for the majority of subjects following corticosteroid treatment. A subanalysis of the primary endpoint in those subjects from the ITT population reporting at least one allergy diagnosis in the medical history captured at screening, showed that FeNO fell by − 50,6% (*p* < 0.00001). This indicates that the population for the most part had allergic asthma and that the FeNO change after corticosteroids was similar to the ITT population as a whole.

**Table 3:** The change in asthma outcomes from V1 to V2 for the ITT population

FeNO (ppb)	Visit 1	Visit 2	Change V1 to V2
n	84	84	84
Mean	81.13	39.42	−41.71
s.d.	48.748	24.191	45.058
% change from baseline	–	–	−51.4
*p*-value	–	–	<.0001
**ACQ6/pACQ6**			
n	84	84	84
mean	2.056	1.044	−1.000
s.d.	1.033	0.6205	1.1264
p-value			<.0001
**FEV**_**1**_**(L)**			
n	84	84	84
mean	2.676	2.883	0.207
s.d.	0.9115	0.8926	0.3518
p-value			<.0001
**TASX**			
n	81	81	81
Mean	4.43	3.03	−1.40
s.d.	3.80	3.06	2.22
p-value	–		< 0.0001

### Secondary outcomes

**Fig. 4:**
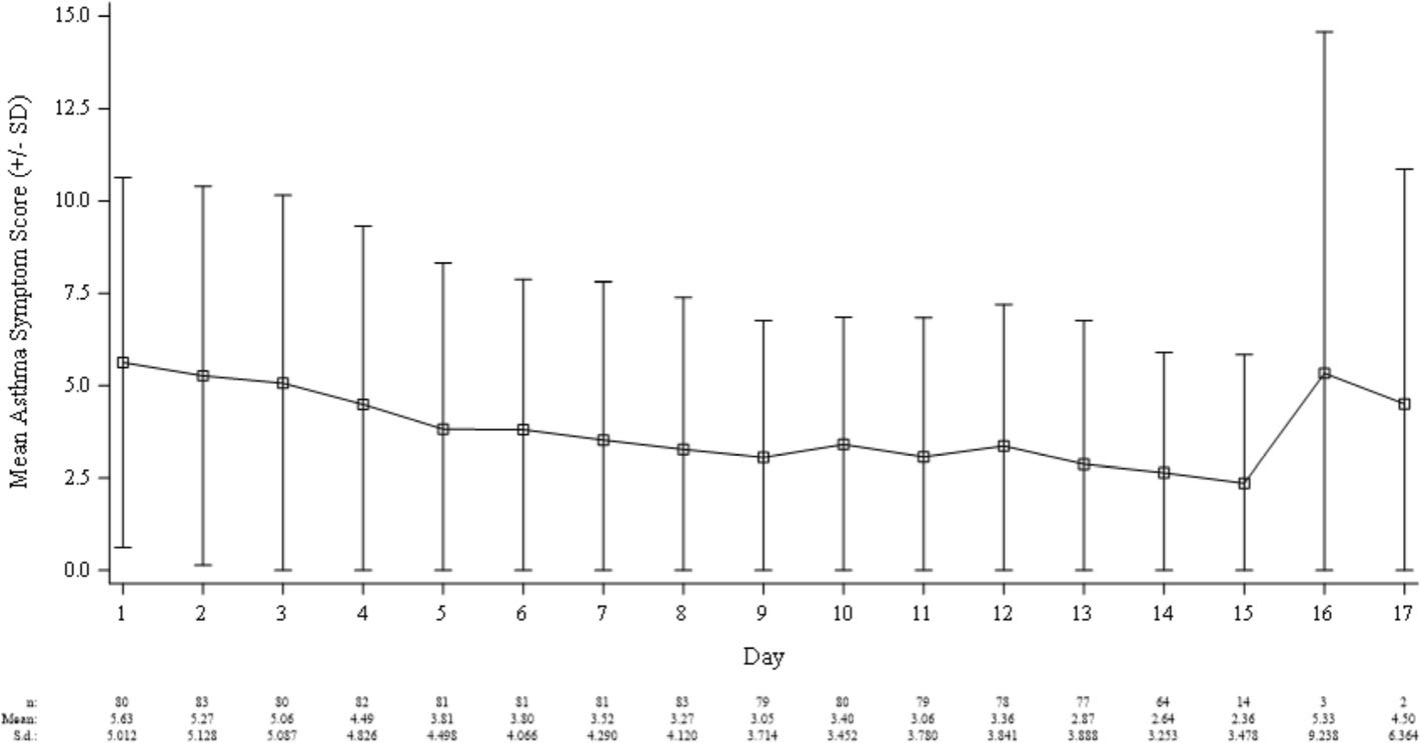
The total asthma symptom scores by study day. The plot includes 1 standard deviation error bars (limited on the lower bound to a value of 0.0 so as to avoid negative values) and notes the sample size (N,) mean, and SD of each time point. Of importance, Days 15, 16, and 17 (the allowable window of 3 days beyond Visit 2) includes a small number of observations at *N* = 14, 3, and 2, respectively (denoted by separate boxed area in the plot). In general, asthma symptom scores decrease from Days 1–15. Due to low N, the estimates for Days 16 and 17 do not provide confidence in any trend

There were highly significant improvements in FEV_1_, ACQ6/pACQ6 and TASX (Table 3) from Visit 1 to Visit 2. The change in TASX for the ITT population is shown in Fig. 4. Of note, the values at Days 15–17 vary considerably due to the fact that few subjects (*N* = 14, 3, and 2, respectively) continued treatment out to these time points, which were within the allowable window of 3 days beyond Visit 2.

### Correlation between changes in asthma domains

Table 4 reports a matrix for simple Pearson correlations for the absolute changes in FeNO and other secondary measures as well as the % changes in FeNO and other measures. The following comparisons showed modest correlation: absolute and % changes in FeNO vs changes in ACQ6/pACQ6 (r values in the range 0.24–0.25; *p* values 0.0280 and 0.0203, respectively), absolute changes in ACQ6 vs FEV_1_ (r = − 0.21, *p* = 0.0580), and absolute and % changes in ACQ6 vs TASX (r values in the 0.29–0.35, p values 0.0015 and 0.0105, respectively).

**Table 4:** Correlation between changes from V1 to V2 in asthma domains for the combined adult and pediatric ITT population

	Change FEV_1_ L	Change FeNO ppb	Change ACQ6	Change TASX
Change FEV_1_ L				
Change FeNO ppb	−0.14 *P* = 0.2161			
Change ACQ6	−0.21 P = 0.0580	0.24 ***P*** **= 0.0280**		
Change TASX	−0.06 *P* = 0.5843	0.13 *P* = 0.2571	0.35 ***P*** **= 0.0015**	
	% Change FEV_1_	% Change FeNO	% Change ACQ6	% Change TASX
% Change FEV_1_				
% Change FeNO	−0.09 *P* = 0.4210			
% Change ACQ6	−0.14 *P* = 0.2057	0.25 ***P*** **= 0.0203**		
% Change TASX	0.03 *P* = 0.7740	0.12 *P* = 0.3209	0.29 ***P*** **= 0.0105**	

### Concordance/discordance for the combined ITT population

Concordance/discordance were assessed between each pair of asthma domains for any change around 0 (Table 5) and for changes around the MID (Table 6) for the combined adult and pediatric ITT population. Table 7 (Panel A) presents the data in descending order for the concordance between pairs of domains for changes around 0. It can be seen that FeNO and ACQ6 have the highest concordance whereas FEV_1_ versus ACQ6 and TASX occupy the 2 lowest concordance values.

**Table 5:** Concordance (in bold text) and discordance for changes between pairs of asthma domains from V1 to V2 for any change around 0 **for the combined adult and pediatric PP population**

Absolute values		FEV_1_ change		ACQ6 change		TASX change	
		Improve	Worsen	Improve	Worsen	Improve	Worsen
FeNO change	Improve	**54 (65.9%)**	19 (23.2%)	**62 (75.6%)**	11 (13.4%)	**54 (66.7%)**	18 (22.2%)
	Worsen	4 (4.9%)	**5 (6.1%)**	6 (7.3%)	**3 (3.7%)**	6 (7.4%)	**3 (3.7%)**
FEV1 change	Improve			**48 (58.5%)**	10 (12.2%)	**42 (51.9%)**	16 (19.8%)
	Worsen			20 (24.4%)	**4 (4.9%)**	18 (22.2%)	**5 (6.2%)**
ACQ6 change	Improve					**53 (65.4%)**	14 (17.3%)
	Worsen					7 (8.6%)	**7 (8.6%)**

**Table 6:** Concordance (in bold text) and discordance for changes between pairs of asthma domains from V1 to V2 for changes equal or greater than the MID (a subset of the PP population). For FeNO, a change of 10 ppb was used (ATS, 2011) whereas for TASX, changes around 0 were used as no MID is available

Absolute values		FEV_1_ change		ACQ6 change		TASX change	
		Improve	Worsen	Improve	Worsen	Improve	Worsen
FeNO change	Improve	**25 (80.7%)**	6 (19.4%)	43 (93.5%)	2 (4.4%)	45 (75.0%)	13 (21.7%)
	Worsen	0 (0.0%)	**0 (0.0%)**	1 (2.2%)	0 (0.0%)	1 (1.7%)	1 (1.7%)
FEV_1_ change	Improve			22 (71.0%)	2 (6.5%)	26 (56.5%)	10 (21.7%)
	Worsen			6 (19.4%)	1 (3.2%)	8 (17.4%)	2 (4.4%)
ACQ6 change	Improve					43 (75.4%)	9 (15.8%)
	Worsen					1 (1.8%)	4 (7.0%)

**Table 7:** Concordance/discordance for pairs of asthma domains considering changes from V1 to V2 considering any change > 0 and changes at < or > than the MID level for the combined adult and pediatric ITT population. The Table is organized in descending order for the data for changes around 0

Asthma domain	Panel A (ITT population)	Panel B (PP population)
	Changes > or < 0 Concordance/discordance (%)	Changes > of < MID Concordance/discordance (%)
FeNO and ACQ6	**79.3/20.7**	**93.5/6.6**
ACQ6 and TASX	74.0/25.9	82.4/17.6*
FeNO and FEV1	71.9/28.1	80.7/19.4
FeNO and TASX	70.4/29.6	76.7/23.3*
FEV1 and ACQ6	63.4/36.6	74.2/25.9
FEV1 and TASX	58.1/41.9	60.9/39.1*

#### Changes around MID

In Table 7 Panel B, it can be seen that concordance for changes around the MID for all comparisons increases and the order is the same as for changes around 0. The concordance between pairs of domains was still highest for FeNO and ACQ6 with changes between ACQ6 and TASX, both symptom assessment instruments second highest. FEV_1_ was the most discordant when assessed against ACQ6 and TASX but demonstrated the third highest concordance with FeNO for both changes around 0 and the MID. Of note, only 17/82 subjects demonstrated concordant MID changes for improvement for the three variables FEV_1_, ACQ6 and FeNO (data not shown).

### Concordance/discordance for the adults and pediatric ITT populations separately

When considering the adult and pediatric populations separately, the findings were similar to those presented for the combined ITT populations (data shown in Table 8, panels A and B and supplemental Tables E1-E4). Again, when moving from changes around 0 to MID changes, there was a universal increase in concordance (Table 8). For changes around 0, for adults alone, FEV_1_ concordance with TASX and ACQ6 remained the lowest, and FeNO vs ACQ6 was still the highest. For the pediatric population, FEV_1_ vs TASX was still the lowest concordance, but ACQ6 and TASX had the highest concordance. For changes around the MID, TASX vs ACQ6 was the highest and FEV_1_ vs TASX the lowest for adults and children.

**Table 8:** Concordance/discordance for pairs of asthma domains considering changes from V1 to V2 considering any change around 0 (ITT population) and changes at the MID level (PP population) for the adult (Panel A) and pediatric (Panel B) populations. The Table is organized in descending order for the data for changes around 0

Asthma domain	Changes > or < 0 Concordance/discordance (%)	Changes > of < MID Concordance/discordance (%)
**Panel A (adult ITT population)**		
FeNO and ACQ6	**82.2/17.7**	**96.2/3.9**
FeNO and TASX	72.7/27.3	75.0/25.0
ACQ6 and TASX	68.2/31.8	74.2/25.8
FeNO and FEV_1_	66.6/33.4	76.2/23.8
FEV_1_ and TASX	59.1/40.9	61.3/38.7
FEV_1_ and ACQ6	57.8/42.3	81.0/19.1
**Panel B (pediatric population)**		
ACQ6 and TASX	**81.1/18.9**	**92.3/7.7**
FeNO and FEV_1_	78.4/21.6	90.0/10.0
FeNO and ACQ6	75.7/24.3	90.0/10.0
FEV_1_ and ACQ6	70.3/29.7	60.0/40.0
FeNO and TASX	67.6/32.4	78.6/21.5
FEV_1_ and TASX	56.8/43.2	60.0/40.0

## Discussion

We report for the first time the degree of concordance between dynamic changes in asthma domain measures for airway inflammation (FeNO), lung function (FEV_1_), asthma control (ACQ6/pACQ6) and daily symptoms (TASX) in steroid naïve adults and children after approximately 2 weeks of corticosteroid therapy. Of note, change in FeNO showed the highest concordance with change in ACQ6 for the combined ITT, adult, and pediatric populations for changes around 0 and for changes around the MID. In general, change in FEV_1_ demonstrated lowest concordances versus change in the other domains with the exception of FeNO. Of note, only 17 of 82 subjects demonstrated concordant ACQ6, FEV_1_, and FeNO changes reflecting improvement.

Based on the findings, the message is clear that the asthma domains of airway inflammation, symptoms, asthma control and lung function demonstrate significant discordance for changes over time. Mechanistically, there is of course a relationship between these domains. Airway inflammation, regarded as a primary driver can worsen airflow limitation, and bronchial hyperresponsiveness, and produce symptoms e.g. wheezing, chest tightness, and dyspnea. Airway inflammation also increases during the night, and this can account for nocturnal asthma. Asthma control measures such as ACQ6 reflect a constellation of symptoms, and recue medication use. A comprehensive assessment of asthma at any timepoint therefore requires the measurement of asthma symptoms and control, lung function, and airway inflammation.

The high concordance of FeNO with ACQ6 suggests that FeNO may be a primary driver of other asthma domains, i.e. airway inflammation drives changes in other domains. The ACQ6 includes 5 symptom questions (items 1–5), as well as rescue medication use (item 6) and thus includes two asthma domains, taking rescue use as a separate domain. ACQ6 and TASX also show high concordance which is predictable as both assess asthma symptoms. The relatively low concordance of FEV_1_ with ACQ6 and TASX may be related to a component of fixed airflow obstruction or other reasons. However, FeNO and FEV_1_ demonstrate higher concordance which might be explained by airway wall inflammation causing worsening in airflow obstruction due to edema, mucus gland hypertrophy and smooth muscle hyperresponsiveness.

The lack of concordance or tight correlation between these domains can be explained in several ways. First, there may be heterogeneous degrees of abnormality for each domain present in individual patients at a single time point and there is a correlation between the change in a domain and the degree of abnormality in that domain (room for change). For example, one patient may have significant airway inflammation but low symptomatology, either because they have yet to experience deteriorating lung function, or they may be poor perceivers and are not sensitive to their lung function decline. Conversely, another patient with marked symptoms may not have elevated airway inflammation if their symptoms are due to fixed reduction in lung function due to airway remodeling.

Second, when patients receive corticosteroids or other interventions as in the study reported here, there may be disparate time courses for change of the individual components. In a 2-week study as was done here, the change in FeNO is probably complete, but symptoms, asthma control and lung function may still be improving and may not be at a steady state. The FEV_1_ response to ICS in particular may still be improving many weeks later.

Third, the development of airway remodeling in certain patients over many years may lead to persistent airflow obstruction (low FEV_1_) that does not respond to a significant degree, whereas FeNO and ACQ6 respond. Conversely, lung function can be near normal in some subjects, especially children, and thus may not have room for improvement.

Finally, one must also consider the intrinsic limitations of all domain measures. FeNO measures one proinflammatory mediator and cannot reflect the entire complexity of airway inflammation present in an individual patient. Indeed, some of the subjects may have had neutrophilic inflammation with or without eosinophilic inflammation, which may not have responded to corticosteroids. FEV_1_ is less sensitive to small airway airflow limitation and cannot reflect airflow limitation throughout the airway tree. ACQ6/pACQ6 while well validated may not capture the entire asthma experience.

The asthma symptom questionnaire (ASX) was used here for the first time and is yet to be validated as a patient reported outcome but seems to show promise demonstrating high concordance with ACQ6 in the combined and separate adult and pediatric populations but lower concordance with FEV_1_. This is perhaps to be expected as both ACQ6 and ASX are symptom domains.

The lack of perfect concordance between asthma domains has been observed in the literature. Kaminski, et al. studied the impact of attendance at a 1-week summer camp on asthma domains in children with asthma [8]. At the end of camp, FeNO showed a decrease of 45% (*p* < 0.0001) whereas ACQ7 only fell by 14% (*p* = 0.72) and mean FEV_1_% predicted remained unchanged.

Khalili et al. reported no significant association was found between FeNO level and several measures of asthma control including the ACQ7, and the Asthma Control Test (ACT) [9]. Quadvlieg, et al. reported a similar lack of correlation between FeNO and ACQ7 in 134 patients with asthma [10]. Patients were divided into the three groups according to their level of asthma control determined by ACQ7 [well-controlled asthma (ACQ7 score < 0.75), borderline (0.75 < ACQ7 score < 1.5) and uncontrolled asthma (ACQ7 score ≥ 1.5)]. There were no significant differences in the FeNO levels between the groups.

Thomas et al. evaluated FeNO, lung function, ACQ7, and the Royal College of Physicians 3 questions (RCP scores) in 35 adults and children with asthma at 2-week intervals over 12 weeks [11]. Weak or absent correlations existed with lung function and there were no correlations with FeNO levels. In a report by Obase, et al. [12], during step down or continuation of inhaled corticosteroid therapy, FeNO levels increased or decreased, respectively, without change in lung function or symptoms assessed by ACQ7. The authors conclude that caution is needed when stepping down corticosteroid therapy solely based on symptoms or lung function. The Airways Disease Endotyping for Personalized Therapeutics (ADEPT) study evaluated FeNO and multiple asthma characteristics in 157 adult asthmatics of varying severity [13]. FeNO did not demonstrate any significant or meaningful associations with lung function or ACQ7. In a metanalysis of the efficacy of anti-interleukin-13 therapies in asthma, Luo, et al. reported that treatment significantly improved peak expiratory flow (PEF), decreased FeNO and asthmatic exacerbation rate, but without decrease in blood or sputum eosinophil levels, improvement in FEV_1_, or reduction in ACQ7 scores [14]. In a study of the effects of exercise and/or diet on asthma control in 125 obese asthmatic patients, Toennesen reported that in the group receiving both interventions, ACQ7 fell significantly compared to either intervention alone, but there were no significant changes in FEV_1_ or ACQ7 in any of the groups [15].

In addition to the concordance reported here for changes in asthma domains, others have noted the low concordance between domains at a single time point [16, 17]. If asthma domains display discordance at a single timepoint, it is logical that changes in these parameters over time would be similar.

Weaknesses of our study include the absence of objective measures of compliance with corticosteroid therapy, although self-reported compliance was very high. Additionally, while there are established MIDs for ACQ6 and FEV_1_, for FeNO there is no established MID and therefore we used the ATS definition of a meaningful change from 2011 of 10 ppb [7] though this might be suboptimal. In addition, the MIDs we used may not themselves be universally applicable e.g. for children.

## Conclusion

In conclusion, while there is substantial concordance between changes in the asthma domains following short-term corticosteroid therapy, there is moderate discordance especially between FEV_1_ and other domains. The findings highlight the complex relationships between asthma domains and emphasize the wisdom of assessing multiple parameters in order to fully understand an individual patient’s asthma.

## Supplementary information

**Additional file 1: Table E1**. Concordance (bolded) and discordance for changes between pairs of asthma domains from V1 to V2 for any change around zero (**adults only**) for the PP population. **Table E2**. Concordance (bolded) and discordance for changes between pairs of asthma domains from V1 to V2 for changes equal or greater than the MID (**adults only**). For FeNO, a change of 10 ppb was used (ATS, 2011) whereas for TASX, changes around 0 were used as no MID is available for the PP population. **Table E3**. Concordance (bolded) and discordance for changes between pairs of asthma domains from V1 to V2 for any change around zero (**pediatric subjects only**) for the PP population **Table E4**. Concordance (bolded) and discordance for changes between pairs of asthma domains from V1 to V2 for changes equal or greater than the MID (**pediatric subjects only**) for the PP population. For FeNO, a change of 10 ppb was used (ATS, 2011) whereas for TASX, changes around 0 were used as no MID is available.

## Data Availability

The datasets generated and/or analyzed during the current study are not publicly available as these are propriety but are available from Spirosure Inc. on reasonable request.

## Abbreviations

FeNO: Fractional concentration of exhaled nitric oxide
FEV_1_: Forced expired volume in one second
ACQ6 or ACQ7: Asthma control questionnaire (6 item or 7-item)
ASX: Asthma symptom questionnaire
TASX: Total score from the ASX
AM: Morning
PM: Evening
C: Concordance
D: Discordance
C.D: Concordance/discordance
MID: Minimal important difference (clinical)
ICS: Inhaled corticosteroids
OCS: Oral corticosteroids
SABA: Short-acting bronchodilator
ATS: American Thoracic Society
V: Visit
ITT: Intent to Treat
RCP: Royal College of Physicians
ADEPT: Airways Disease Endotyping for Personalized Therapeutics
PEF: Peak Expiratory Flow
IgE: Immunoglobulin E
