# Supplementary material for: Concordance for changes in allergic asthma domain variables after short-term corticosteroid therapy
Source: BMC Pulm Med. 2020 May 14;20:139. doi: 10.1186/s12890-020-1166-2 (PMC7222465; doi:10.1186/s12890-020-1166-2)
Supplement: Supplementary file 1 — Additional file 1: Table E1. Concordance (bolded) and discordance for changes between pairs of asthma domains from V1 to V2 for any change around zero (adults only) for the PP population. Table E2. Concordance (bolded) and discordance for changes between pairs of asthma domains from V1 to V2 for changes equal or greater than the MID (adults only). For FeNO, a change of 10 ppb was used (ATS, 2011) whereas for TASX, changes around 0 were used as no MID is available for the PP population. Table E3. Concordance (bolded) and discordance for changes between pairs of asthma domains from V1 to V2 for any change around zero (pediatric subjects only) for the PP population Table E4. Concordance (bolded) and discordance for changes between pairs of asthma domains from V1 to V2 for changes equal or greater than the MID (pediatric subjects only) for the PP population. For FeNO, a change of 10 ppb was used (ATS, 2011) whereas for TASX, changes around 0 were used as no MID is available. [file 12890_2020_1166_MOESM1_ESM.docx]

**Table E1**: Concordance (bolded) and discordance for changes between pairs of asthma domains from V1 to V2

for any change around zero (**adults only**) for the PP population

| Absolute values |  | FEV_1_ change | | ACQ6 change | | TASX change | |
| --- | --- | --- | --- | --- | --- | --- | --- |
|  |  | Improve | Worsen | Improve | Worsen | Improve | Worsen |
| FeNO change | Improve | **29 (64.4%)** | 12 (26.7%) | **35 (77.8%)** | 6 (13.3%) | **29 (65.9%)** | 11 (25.0%) |
|  | Worsen | 3 (6.7%) | **1 (2.2%)** | 2 (4.4%) | **2 (4.4%)** | 1 (2.3%) | **3 (6.8%)** |
| FEV_1_ change | Improve |  |  | **25 (55.6%)** | 7 (15.6%) | **22 (50.0%)** | 10 (22.7%) |
|  | Worsen |  |  | 12 (26.7%) | **1 (2.2%)** | 8 (18.2%) | **4 (9.1%)** |
| ACQ6 change | Improve |  |  |  |  | **26 (59.1%)** | 10 (22.7%) |
|  | Worsen |  |  |  |  | 4 (9.1%) | **4 (9.1%)** |

**Table E2**: Concordance (bolded) and discordance for changes between pairs of asthma domains from V1 to V2

for changes equal or greater than the MID **(adults only)**. For FeNO, a change of 10 ppb was used (ATS, 2011) whereas for TASX, changes around 0 were used as no MID is available for the PP population

| Absolute values |  | FEV_1_ change | | ACQ6 change | | TASX change | |
| --- | --- | --- | --- | --- | --- | --- | --- |
|  |  | Improve | Worsen | Improve | Worsen | Improve | Worsen |
| FeNO change | Improve | **16 (76.2%)** | 5 (23.8%) | **25 (96.2%)** | 0 (0.0%) | **23 (71.9%)** | 8 (25.0%) |
|  | Worsen | 0 (0.0%) | **0 (0.0%)** | 1 (3.9%) | **0 (0.0%)** | 0 (0.0%) | **1 (3.1%)** |
| FEV_1_ change | Improve |  |  | **16 (76.2%)** | 1 (4.8%) | **17 (54.8%)** | 7 (22.6%) |
|  | Worsen |  |  | 3 (14.3%) | **1 (4.8%)** | 5 (16.1%) | **2 (6.5%)** |
| ACQ6 change | Improve |  |  |  |  | **22 (71.0%)** | 7 (22.6%) |
|  | Worsen |  |  |  |  | 1 (3.2%) | **1 (3.2%)** |
|  | | | | | | | |

**Table E3**: Concordance (bolded) and discordance for changes between pairs of asthma domains from V1 to V2

for any change around zero **(pediatric subjects only)** for the PP population

| Absolute values |  | FEV_1_ change | | ACQ6 change | | TASX change | |
| --- | --- | --- | --- | --- | --- | --- | --- |
|  |  | Improve | Worsen | Improve | Worsen | Improve | Worsen |
| FeNO change | Improve | **25 (67.6%)** | 7 (18.9%) | **27 (73.0%)** | 5 (13.5%) | **25 (67.6%)** | 7 (18.9%) |
|  | Worsen | 1 (2.7%) | **4 (10.8%)** | 4 (10.8%) | **1 (2.7%)** | 5 (13.5%) | **0 (0.0%)** |
| FEV_1_ change | Improve |  |  | **23 (62.2%)** | 3 (8.1%) | **20 (54.1%)** | 6 (16.2%) |
|  | Worsen |  |  | 8 (21.6%) | **3 (8.1%)** | 10 (27.0%) | **1 (2.7%)** |
| ACQ6 change | Improve |  |  |  |  | **27 (73.0%)** | 4 (10.8%) |
|  | Worsen |  |  |  |  | 3 (8.1%) | **3 (8.1%)** |

**Table E4**: Concordance (bolded) and discordance for changes between pairs of asthma domains from V1 to V2

for changes equal or greater than the MID **(pediatric subjects only)** for the PP population. For FeNO, a change of 10 ppb was used (ATS, 2011) whereas for TASX, changes around 0 were used as no MID is available.

| Absolute values |  | FEV_1_ change | | ACQ6 change | | TASX change | |
| --- | --- | --- | --- | --- | --- | --- | --- |
|  |  | Improve | Worsen | Improve | Worsen | Improve | Worsen |
| FeNO change | Improve | **9 (90.0%)** | 1 (10.0%) | **18 (90.0%)** | 2 (10.0%) | **22 (78.6%)** | 5 (17.9%) |
|  | Worsen | 0 (0.0%) | **0 (0.0%)** | 0 (0.0%) | **0 (0.0%)** | 1 (3.6%) | **0 (0.0%)** |
| FEV_1_ change | Improve |  |  | **6 (60.0%)** | 1 (10.0%) | **9 (60.0%)** | 3 (20.0%) |
|  | Worsen |  |  | 3 (30.0%) | **0 (0.0%)** | 3 (20.0%) | **0 (0.0%)** |
| ACQ6 change | Improve |  |  |  |  | **21 (80.8%)** | 2 (7.7%) |
|  | Worsen |  |  |  |  | 0 (0.0%) | **3 (11.5%)** |
|  | | | | | | | |
